# Sensor-on-Microtips: Design and Development of Hydrothermally Grown ZnO on Micropipette Tips as a Modified Working Electrode for Detection of Glucose

**DOI:** 10.3390/mi14030498

**Published:** 2023-02-21

**Authors:** Priyannth Ramasami Sundhar Baabu, Ganesh Kumar Mani, John Bosco Balaguru Rayappan, Yuichiro Tsuyuki, Toshiyuki Inazu, Kazuyoshi Tsuchiya

**Affiliations:** 1 School of Chemical and Biotechnology (SCBT), SASTRA Deemed University, Thanjavur 613 401, India; 2 Department of Physics, Korea Advanced Institute of Science and Technology (KAIST), Daejeon 34141, Republic of Korea; 3 Micro/Nano Technology Center, Tokai University, Hiratsuka 259-1292, Japan; 4 Centre for Nanotechnology & Advanced Biomaterials (CeNTAB), SASTRA Deemed University, Thanjavur 613 401, India; 5 Hasegawa Machinery Limited, 307 Matsuoka, Fuji-shi 416-0909, Japan; 6 Department of Applied Chemistry, School of Engineering, Tokai University, Hiratsuka 259-1292, Japan

**Keywords:** microtips, working electrode, hydrothermal growth, ZnO nanostructures, glucose detection

## Abstract

Miniaturization of electrochemical components has become less common in the last decade, with the focus predominantly being the design and development of state-of-the-art microelectrodes for achieving small volume analysis of samples. However, such microelectrodes involve cumbersome processing procedures to convert the base material for the required application. A potential paradigm shift in such miniaturization could be achieved by using cheaper alternatives such as plastics to build electrochemical components, such as micropipette tips made of polypropylene, which are commercially available at ease. Hence, this work presents the design of an electrochemical working electrode based upon a micropipette tip, involving minimal processing procedures. Furthermore, such a working electrode was realized by sputtering silver onto a bare micropipette tip using a radio-frequency sputtering technique, to obtain electrical contacts on the tip, followed by hydrothermal growth of ZnO, which acted as the active electrode material. The ZnO nanostructures grown on the micropipette tip were characterized for their morphology and surface properties using a scanning electron microscope (SEM), laser microscope, Raman spectrometer, and X-ray photoelectron spectrometer (XPS). The developed micropipette tip-based electrode was then used as the working electrode in a three-electrode system, wherein its electrochemical stability and properties were analyzed using cyclic voltammetry (CV). Furthermore, the above system was used to detect glucose concentrations of 10–200 µM, to evaluate its sensing properties using amperometry. The developed working electrode exhibited a sensitivity of 69.02 µA/µM cm^−2^ and limit of detection of 67.5 µM, indicating the potential for using such modified micropipette tips as low-cost miniaturized sensors to detect various bio-analytes in sample solutions.

## 1. Introduction

Electrochemical measurement has become central to the development of sensors that are highly selective and sensitive, possess a broad linear range of detection, and allow ease of handling and portability [1,2]. Furthermore, electrochemical systems offer a degree of miniaturization of their components, so that the analyses performed permit small volume analysis, possible measurement in the absence of an electrolyte, enhancements in the signal-to-noise ratio, and coherent mass transport [3,4,5]. Diverse materials have been tried for the manufacture of miniaturized electrochemical components, including metals such as gold, silver, and platinum in the form of microelectrodes [6,7,8,9]. However, the fabrication of such microelectrodes involves cumbersome and expensive processing techniques.

A potential paradigm shift could be the use of cheaper alternatives like plastics to build miniaturized electrochemical components, such as in micropipette tips that are predominantly made of polypropylene and commercially available. A research field has developed exclusively dedicated to using miniaturized components for analysis, called lab-on-a-tip. To begin with, atomic force microscopy (AFM) tips were used for this purpose [10]. However, due to the high costs and extensive and time-consuming processing techniques involved, they are slowly being replaced by micropipette tips. Several works have focused on developing electrochemical systems using micropipette tips, where a three-electrode system is integrated onto a single micropipette tip [11,12]. Despite their superior design integration, such systems have not been applied for biosensing.

On the other hand, novel nanostructured materials that possess idiosyncratic properties for various applications have proven their utility in the electrochemical detection of bio analytes, playing the role of catalysts or mediators [13]. Of the many materials that have been reported so far, semiconducting metal oxide nanostructures have garnered much attention, owing to their tunable properties, predominantly in relation to their stoichiometry, band gap, and morphology [14]. Zinc oxide (ZnO) nanostructures, generally with a hexagonal wurtzite structure, are materials with a direct and wide band gap (3.37 eV) [15]. In addition, ZnO offers the requisite electrochemical properties, such as faster electron transfer and larger reaction surface coverage [16,17,18,19]. These properties were the primary reason behind our choosing to grow ZnO nanostructures on a micropipette tip, to act as a modified working electrode.

Chemical methods to synthesize ZnO nanostructures are plentiful. Hydrothermal [20], chemical vapor deposition [21], sol-gel [15], and precipitation [22] are some of the commonly used methods. Since large yields of ZnO nanostructures with uniform morphology and properties are required for our objective of developing modified micropipette tips that could act as working electrodes, hydrothermal method was employed, owing to its controllable crystal growth mechanism and reaction kinetics. Parameters such as the pressure, temperature, and vapor pressure inside the hydrothermal chamber, viscosity, dielectric constant and surface tension of the water as the reaction proceeds, and diffusion coefficients of the precursor molecules are essential in realizing the repeatable synthesis of ZnO nanostructures that is required for developing modified micropipette tips into electrodes [23].

Glucose, being one of the most widely used biomolecules in developing and demonstrating the working of biosensors, was used in our study, to allow our modified micropipette tip to act as a working electrode, while also possessing the ability to function as a biosensor. The significance of detecting glucose in bodily fluids has been reported in over a million research articles, utilizing various strategies [24,25,26,27,28,29,30,31,32], reviews [13,33,34], and book chapters [35,36,37,38].

Hence, in our present work, hydrothermal method was employed to grow ZnO nanostructures over a micropipette tip, which could subsequently be used as a working electrode in a three-electrode electrochemical system. An electrochemical biosensor making use of a micropipette tip as one of its electrodes has not previously been proposed in the literature, and hence this represents the novelty of our work. Acknowledging the few reports on integrating a three-electrode system onto a micropipette tip, which did not use their system for biosensing, we herein demonstrate a proof-of-concept for the design and development of modified micropipette tip-based biosensors for glucose detection. These modified micropipette tips were prepared through radio frequency sputtering of Ag, followed by hydrothermal growth of ZnO nanostructures over the surface. Our work couples the field of lab-on-a-tip with biosensors, providing a cheap and unique alternative to the conventional disk electrodes used for biosensing.

## 2. Materials and Methods

### 2.1. Chemicals Used

Zinc nitrate hexahydrate (Zn(NO_3_)2·6H_2_O, 99.9%, Wako Chemicals—265-01032, Richmond, VA, USA), hexamethyltetramine (HMTA) (C_6_H_12_N_4_, 99%, Wako Chemicals—085-00335), sodium chloride, and D(+)-Glucose (Wako Pure Chemical Industries, Osaka, Japan) were utilized in this study. All reagents and solutions were prepared using deionized water.

### 2.2. Sputtering of Ag on Bare Micropipette Tips as Electrical Contacts

**Figure 1:**
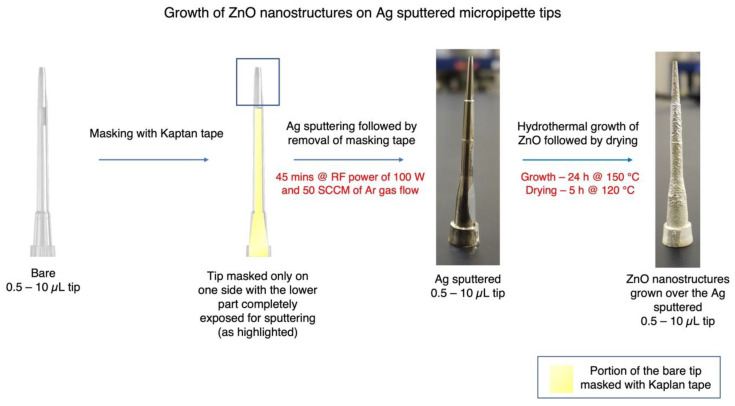
Illustration of the stages of modification of the bare micropipette tips (0.5–10 µL), starting from masking with Kapton tape, with only one side covered, leaving the other side and lower part completely exposed for sputtering. Subsequently, RF sputtering of Ag was performed for 45 min, followed by removing the masking tape. Finally, ZnO nanostructures were grown over the Ag-sputtered micropipette tips for 24 h at 150 ℃, followed by drying for 5 h at 120 ℃ and several rinsing steps.

Considering the fact that the polypropylene micropipette tips of 0.5–10 µL (Eppendorf, inner diameter, ∅~0.33 mm) were not electrically conductive, a thin film of Ag was sputtered onto the tips using a radio frequency (RF) sputtering technique, as seen in Figure 1. Before performing the coating, the tips were masked with Kapton tape, except for the rear tip end and a narrow vertical region. The sputtering parameters used for the coating were as follows:

Flow rate of Ar gas—50 SCCM.

RF power—100 W.

Working pressure—2 Pa.

Pre-sputtering time—5 min.

Main sputtering time—45 min.

Source to target distance—60 mm.

### 2.3. Growth of ZnO Nanostructures on the Sputtered Micropipette Tips

First, 50 mM of Zinc nitrate hexahydrate and 50 mM of HMTA were mixed together (each 50 mL volume) in an equimolar fashion. The Ag-sputtered micropipette tips were immersed in the above solution for 1 h, after having removed the Kapton tape. The tips were then taken out of the solution and dried at 120 °C in a drying oven for 1 h. Subsequently, ~75 mL of the equimolar Zinc nitrate hexahydrate and HMTA solution was transferred to a Teflon beaker. The dried tips were then put into the beaker and immersed in the precursor solution. The beaker was then put in a hydrothermal chamber, which was then placed into a drying oven at 150 °C for 24 h. After 24 h, the drying oven was switched off and the chamber was left to cool down. A thick translucent white coating was observed, indicating the growth of ZnO nanostructures, which can be seen in Figure 1. The tip ends were dried, rinsed with deionized water to remove excessive ZnO, and placed in a drying oven at 120 °C for 5 h.

### 2.4. Characterization of ZnO Nanostructures

A 3D laser scanning microscope (Model: VK-X100K, Keyence, Itasca, IL, USA) was used to observe the differences in the surface features of the bare micropipette tips, Ag-sputtered tips, and the ZnO grown tips, along with surface thickness measurements of the sputtered and grown thin films. Morphological analysis of the grown nanostructures was performed using a field emission scanning electron microscope (FE-SEM) (Model: Hitachi, S-4800 SEM, Chiyoda City, Japan). The samples used for SEM analysis were prepared by taking a thin film of the ZnO grown over sputtered Ag film on the tip, followed by subsequent coating on a carbon-coated copper grid. Elemental analysis was performed using energy dispersive X-ray spectroscopy (EDS) integrated with an SEM system. The vibrational mode signatures of the grown nanostructures were studied using a Raman spectrometer (Model: XploRA, Horiba, Kyoto, Japan) in the range of 0–4000 cm^−1^. The binding energy profiles and surface level analysis of elements, along with their bonding characteristics, were obtained with an X-ray photoelectron spectrometer (XPS) (Model: PHI Quantera II, ULVAC-PHI, Chigasaki, Japan).

### 2.5. Electrochemical Analysis

**Figure 2:**
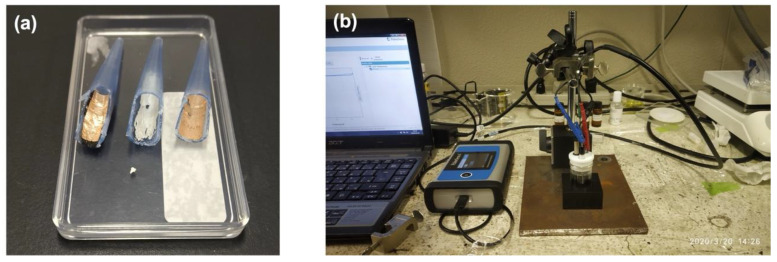
(**a**) Image of the support tip that was cut longitudinally and cross-sectionally, followed by pasting carbon and copper tapes as conductive ends. (**b**) Image showing the electrochemical cell set up for analysis, with the modified tip placed in the support tip.

The electrochemical measurements were performed using a PalmSens4 electrochemical analyzer (PalmSens, Houten, The Netherlands), employing a three-electrode system consisting of a platinum wire as the auxiliary electrode (CHI115, CH Instruments, Bee Cave, TX, USA, 0.5 mm diameter), an Ag/AgCl electrode (CHI115, CH Instruments, USA, 0.5 mm diameter) saturated with 1 M KCl as reference electrode, and the modified micropipette tip as the working electrode. To achieve electrical contact between the developed working electrode using micropipette tips and the crocodile pins of the PalmSens4, the following strategy was employed:

A polypropylene micropipette tip of 1000 µL (Eppendorf, inner diameter, ∅~7 mm) was used as a support for the modified tip to be used as the working electrode in the electrochemical cell. The support tip was cut both longitudinally and cross-sectionally, followed by pasting 5 layers of carbon tape and a layer of copper tape, as shown in Figure 2a.

The modified 0.5–10 µL tip was then inserted into this support tip, to be placed into the electrochemical cell for the analysis, as can be seen in Figure 2b. The conductivity from the rear end of the modified 0.5–10 µL tip to the near end of the support tip in contact with the crocodile pin was checked using a multimeter and was found to be positive every time.

Cyclic voltammetry and amperometry studies were carried out in a 20 mL electrochemical cell with an electrolyte solution of 0.1 M NaCl (15 mL volume). All CV studies were performed at room temperature in a potential window of −0.9 V to +0.9 V. The CV studies were performed in triplicate, for both the Ag-sputtered and ZnO grown over Ag-sputtered modified microtips, to check their reproducibility.

Amperometric sensing studies were carried out at a cathodic potential of −0.6 V for 50 s in triplicate. Varying glucose levels of 10 µM to 10 mM were prepared and studied, to test the sensing performance of the developed sensor using modified micropipette tips during each of the three trials. With regards to the sample preparation for the amperometric studies in each trial, glucose was initially added to the electrolyte solution, followed by stirring the solution at 50 rpm for 1 min for mixing. Amperometric measurements were then performed, without any stirring during the analysis. The solutions used for the CV/amperometry measurement were discarded and the electrochemical cell was cleaned with detergent and dried at room temperature, before use for the next measurement.

## 3. Results and Discussion

### 3.1. Characterization of ZnO-Modified Micropipette Tips

**Figure 3:**
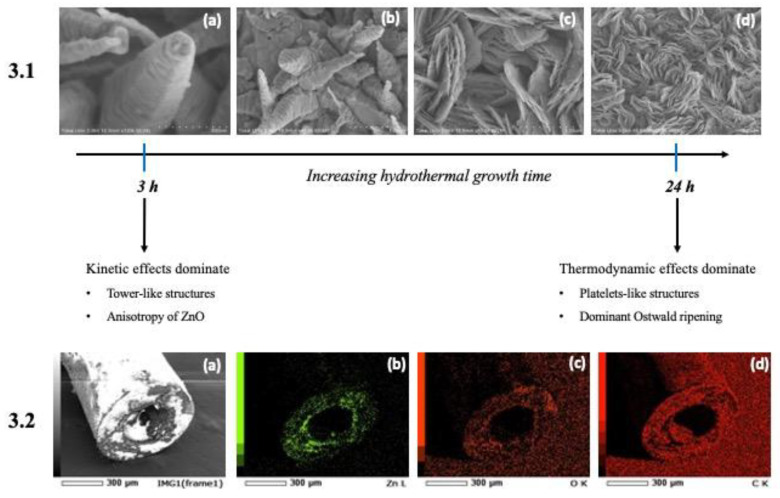
(**3.1 a–d**) SEM images showing the reaction-time-dependent hydrothermal growth of ZnO nanostructures. (**3.2**) Elemental analysis maps representing the distribution of (**b**) Zn, (**c**) O, and (**d**) C elements near the tip end of the micropipette tip (**a**)—represents the region studied for elemental distribution).

The morphological characteristics of the ZnO nanostructures grown on the micropipette tips are shown in Figure 3 (Section 3.1). When the reaction time was 24 h, the hydrothermally grown ZnO exhibited an aggregated platelet-like morphology.

However, from Figure 3 (Section 3.1) it can be observed that, as the reaction time increased, the morphology of the grown ZnO nanostructures also changed significantly. In Figure 3 (Section 3.1 (a)), with a reaction time of ~3 h, tower-like structures of ZnO were obtained as a result of layer-by-layer stacking of the individual nanosheets of ZnO, leading to an asymmetrical width change along the length of the nanostructure. This occurred as a result of the dominant kinetic effects in the hydrothermal chamber, wherein the viscosity of water was significantly reduced, leading to an increased mobility of Zn^2+^ ions, nitrate ions, and ammonia in the solution, thereby possessing greater diffusion coefficients individually. As seen in Figure 3 (Section 3.1 (a,b)), the average height of the tower-like structures was found to be 855 nm, while the width of the visible sides of the hexagonal tower-like structure (from left to right) were found to be 148.87, 215.77, and 123.30 nm, respectively, confirming the asymmetrical width change along the length of the tower-like structure [39].

As seen in the series of equations below (Equations (1)–(4)), during the hydrothermal growth that furnished a high temperature and pressure, the Zn^2+^-amino complex that formed initially when the zinc nitrate and HMTA reacted, underwent thermolysis, due to a shift in equilibrium favoring the backward reaction. In addition, the HMTA and Zn^2+^ ions underwent hydrolysis, resulting in ammonium ions and zinc hydroxide, respectively. The formed zinc hydroxide was then thermally dried to give ZnO.

(1) $$\mathrm{Zn}{\left({\mathrm{NO}}_{3}\right)}_{2}+{\;C}_{6}H_{12}N_{4}\to {\;\mathrm{Zn}}^{2+}\mathrm{amino}\;\mathrm{complex}$$

(2) $$C_{6}H_{12}N_{4}+6H_{2}O\to 6\mathrm{HCHO}+4{\mathrm{NH}}_{3}$$

(3) $${\mathrm{NH}}_{3}+{\;H}_{2}O\to {\;\mathrm{NH}}_{4}{}^{+}+{\;\mathrm{OH}}^{-}$$

(4) $${\mathrm{Zn}}^{2+}+2{\mathrm{OH}}^{-}\to \;\mathrm{Zn}{\left(\mathrm{OH}\right)}_{2}\;\overset{\Delta }{\to }\;\mathrm{ZnO}+{\;H}_{2}O$$

Given this scenario, due to the inherent anisotropic nature of ZnO, crystal planes with a high surface energy would possess a high growth velocity, resulting in differential growth of the various crystal planes, wherein the rapidly growing planes dominated. This domination was possible because of the diffusion ability of the precursor molecules at shorter reaction times in the hydrothermal chamber, explaining the tower-like morphology [15,39].

Furthermore, as the reaction time was increased from 3 h, multiple tower-like ZnO nanostructures aggregated together by virtue of the increasing solubility of ZnO with increasing density, thereby decreasing its diffusion coefficient. Consequently, the probability of collisions between these tower-like structures increased, resulting in a supersaturated solution, where thermodynamic effects dominated. As can be seen from Figure 3 (Section 3.1 (b–d)), Ostwald ripening caused the transition of individual tower-like nanostructures into aggregated platelet-like nanostructures possessing an average width of ~375 nm, when the reaction time reached 24 h [15,39].

**Figure 4:**
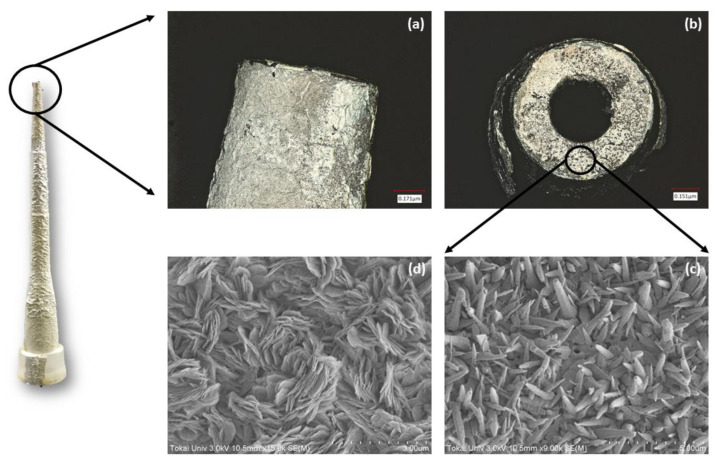
(**a**,**b**) Laser microscope images of the grown ZnO nanostructures over micropipette tip at 10x magnification in different orientations (**c**,**d**) SEM images of aggregated ZnO platelet-like nanostructures at 9 k and 15 k magnifications, respectively.

The elemental analysis maps in Figure 3 (Section 3.2 (a–d)) confirm the presence of significant percentage of zinc and oxygen elements distributed in the tip end of the ZnO grown micropipette tip, with carbon being the dominant element, owing to the bare micropipette tip made of polypropylene. In addition, the laser microscope images in Figure 4a,b reveal the formation of a thin film of ZnO nanostructures grown over the micropipette tip, validating the significant presence of ZnO at the tip end in the elemental analysis, which is essential for sensing purposes.

**Figure 5:**
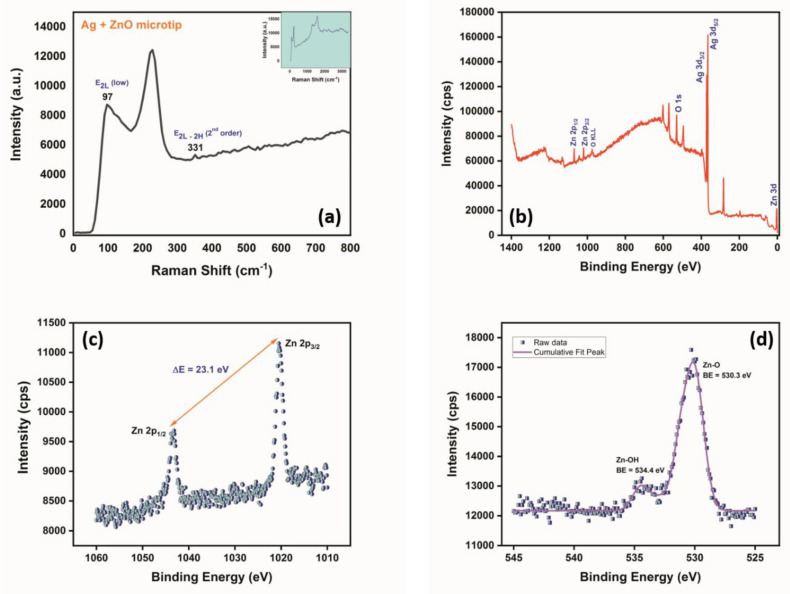
(**a**) Raman spectrum of ZnO grown over a Ag-sputtered micropipette tip between 0–800 cm^−1^ (entire spectrum shown in the inset). (**b**) Survey scan showing the presence of Zn, O, and Ag elements on the surface of the micropipette tip after sputtering and hydrothermal growth. (**c**) Zn 2*p* spectrum revealing the spin orbit doublet of Zn2*p*_1/2_ and Zn 2*p*_3/2_. (**d**) O 1*s* spectra confirming the dominance of Zn-O bonding at the surface over Zn-OH.

The Raman spectrum of the ZnO nanostructures grown over an Ag-sputtered micropipette tip in Figure 5a shows the presence of peaks at 97, 226, and 331 cm^−1^, where the intense peak at 226 cm^−1^ could be attributed to the silver-oxygen mode (Ag-O), due to the fact that the sputtered tip was stored in ambient conditions before hydrothermal growth, resulting in possible oxidation. On the other hand, the peaks at 97 and 331 cm^−1^ correspond to the E_2L_ and 2nd order E_2H_–E_2L_ phonon modes of ZnO [40,41].

From the XPS spectra of the grown ZnO nanostructures shown in Figure 5b–d, the survey spectrum corroborated the presence of Zn and O from the peaks observed at 1020 and 530 eV, respectively. The Zn 2*p* spectrum shown in Figure 5c exhibits a spin orbit doublet at 1043.48 and 1020.38 eV corresponding to Zn 2*p*_1/2_ and Zn 2*p*_3/2_ [15].

The difference in the binding energies between Zn 2*p*_1/2_ and Zn 2*p*_3/2_ was calculated to be 23.10 eV, confirming the +2-oxidation state of Zn. With regards to the XPS spectrum of O 1*s*, the peaks obtained at 534.40 eV and 530.30 eV correspond to Zn-OH and Zn-O, respectively [15]. Hence, from Figure 5d it is clearly evident that ZnO was the dominant form present on the surface of the micropipette tip after modification by sputtering and hydrothermal growth, as corroborated by the ratio of intensities of the two peaks being 1.32 (Zn-O peak intensity/Zn-OH peak intensity).

### 3.2. ZnO Nanostructures Grown on a Micropipette Tip as a Modified Working Electrode

#### 3.2.1. Multilayer Confirmation and Stability Analysis

**Figure 6:**
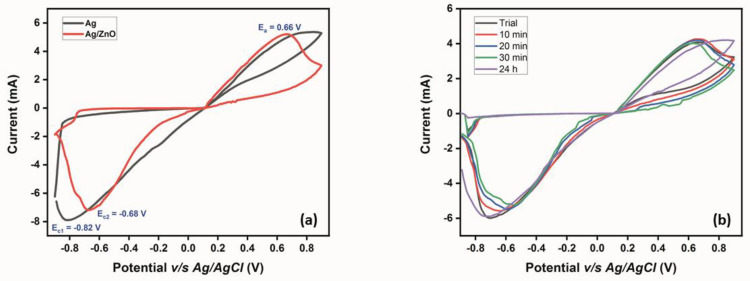
(**a**) CV profile of the Ag-sputtered micropipette tip and ZnO grown over Ag-sputtered micropipette tip, exhibiting a cathodic peak at −0.82 V and redox peaks at 0.66 V and −0.68 V, respectively in 0.1 M NaCl electrolyte at a scan rate of 0.1 V/s. (**b**) Stability profile from CV studies of the ZnO-modified micropipette tip for 24 h and different intervals in between.

Cyclic voltammetry was employed to study and compare the electrochemical properties of the modified micropipette tips: Ag-sputtered microtips and ZnO grown over Ag-sputtered microtips in 0.1 M NaCl electrolyte. As shown in Figure 6a, at a scan rate of 0.1 V/s, the voltammogram for the Ag-sputtered microtip revealed a cathodic peak at −0.82 V with the absence of an anodic peak, while the voltammogram for ZnO grown over the Ag-sputtered microtip exhibited redox peaks at 0.66 and −0.68 V.

When analyzing the CV profile of the Ag-sputtered microtip, interesting features were obtained. When the sweep was performed from −0.9 V to +0.9 V, oxidation of Ag in the presence of chloride ions from the electrolyte occurred weakly, which is why the anodic peak was almost flat. In the reverse sweep, the cathodic peak observed at −0.82 V could be attributed to the reduction of Ag_2_O to Ag, as shown in Equation (5) [42]. Here, it is noted that, in the Raman spectrum, an intense band corresponding to the Ag–O mode was observed, indicating that the Ag-sputtered microtip was oxidized due to its storage in ambient conditions. Hence, the current density of the reduction process was enhanced by the presence of additional Ag_2_O over the tip surface.

(5) $${\mathrm{Ag}}_{2}O\;+{\;H}_{2}O+2e^{-}\to \;2\mathrm{Ag}\;+2{\mathrm{OH}}^{-}$$

Moving the focus of discussion to the ZnO grown over an Ag-sputtered microtip, the anodic and cathodic peaks could be assigned to the redox transition between the oxygen and hydroxide ions. The initial hydroxide source for the oxidation that occurred here possibly came from the hydrated ZnO in the presence of NaCl electrolyte in the forward sweep, followed by the reduction of oxygen liberated in the previous process back to hydroxide ions in the reverse sweep, as shown in Equation (6). It should be noted that bubbles were observed at the counter electrode during this process, corroborating the involvement of oxygen in the redox reaction.

(6) $$O_{2}+2H_{2}O+4{\;e}^{-}\;\to 4{\mathrm{OH}}^{-}\;$$

In addition, the ZnO-modified micropipette tip showed decreased current density and an increased capacitance in comparison to the Ag-sputtered microtip. This was because of the double layer effect, wherein the presence of an additional layer (ZnO, in this case) over an already existing layer (sputtered Ag) increases the double layer capacitance of the system, consequently decreasing the number of electrons diffusing through the double layer and resulting in a decreased current density.

Table 1 reports the significant electrochemical parameters for both the Ag-sputtered microtip- and the ZnO grown over an Ag-sputtered microtip-based working electrodes. From the table, it can be observed that the overpotential for the ZnO-based working electrode (cathodic reaction—0.279 V) was higher than the Ag-sputtered working electrode (cathodic reaction—0.213 V). This is because of the increased diffusion layer thickness in the former’s case, as discussed earlier. This increased overpotential was reflected in their respective Gibbs’ free energy values, with ZnO-based working electrode possessing a greater free energy (~517 kJ/mol) than the Ag-sputtered working electrode (~312 kJ/mol).

Consequently, the equilibrium rate constant for ZnO working electrode was slightly lower than that of the Ag-sputtered working electrode, indicating that the establishment of equilibrium in the former’s case would be slightly delayed compared to the latter. However, the ZnO-based working electrode was chosen for sensing studies, owing to the involvement of four electrons in the sensing mechanism (Equation (6)), resulting in an enhanced current sensitivity over the Ag-sputtered working electrode, which involved only two electrons (Equation (2)).

The stability profile of the modified electrodes was evaluated using CV studies, where the modified microtips were immersed in the electrolyte solution for 10 min, 20 min, 30 min, and 24 h, followed by running a CV at the end of the incubation time. From Figure 6b, it can be observed that the ZnO-modified microtip exhibited significant stability at the intervals chosen for the analysis, with minor variations in the current density and insignificant shifts in potential, at both anodic and cathodic ends. However, the Ag-sputtered tip lacked stability in the chosen electrolyte system, owing to the Ag layer getting peeled away upon removing it from the support microtip, thereby vindicating the use of the ZnO-modified microtip as the working electrode for the sensing studies.

#### 3.2.2. Electrochemical Detection of Glucose Using the ZnO-Modified Micropipette Tip

**Figure 7:**
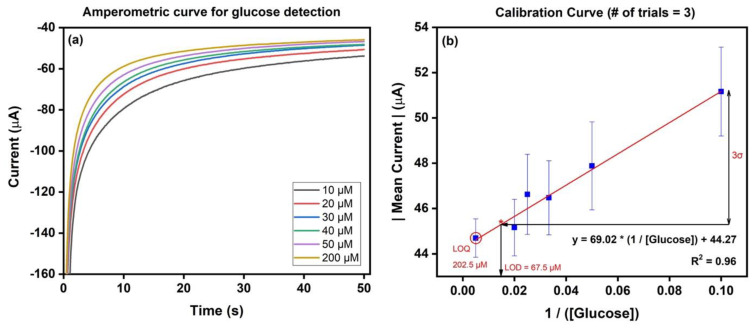
(**a**) Amperometric profile of the ZnO-modified micropipette tip used as a working electrode in 0.1 M NaCl at −0.68 V in the presence of increasing concentrations of glucose (10 µM–10 mM; only the linear range concentrations are represented in the figure). (**b**) Calibration curve for the developed sensor using a ZnO-modified micropipette tip as the working electrode to detect glucose, upon performing three trials (LOD and theoretical LOQ have been represented).

The amperometric profile for the ZnO-modified working electrode with successive addition of 100 µL glucose of increasing concentrations (10 µM–10 mM) in 0.1 M NaCl electrolyte at −0.68 V is given in Figure 7a. Progress to steady-state saturation was observed in ~50 s, owing to the quick detection of glucose, indirectly through the reduction of oxygen to hydroxide ions in the electrolyte solution. From the profile, it is evident that as the concentration of glucose increased, the current density due to the cathodic reaction became saturated. In accordance with our sensing mechanism, where glucose was oxidized to gluconolactone with oxygen being reduced to hydroxide ions and the ZnO nanostructures playing the role of mediator of this electron transfer reaction, the active sites in the nanostructures became filled as the concentration of the analyte (glucose) increased, leading to the observed saturation in the amperometric profile. This resulted in diffusion-limited detection of glucose by our ZnO-modified working electrode, as vindicated by the observed saturation in the current density. Consequently, the calibration curve for glucose detection based on the developed working electrode using ZnO-modified micropipette tips (n = 3) was plotted between the mean current and inverse of glucose concentration, as seen in Figure 7b.

The merits for the developed biosensor include a linear detection range of 10–200 µM, enhanced sensitivity of 69.02 µA µM^−1^ cm^−2^, low detection limit of 67.5 µM, and limit of quantification of 202.5 µM. Table 2 compares the merits of the current work with the available literature. 

The detection limit was estimated by considering the mean current point on the fitted linear line at 3*σ units below the lowest concentration of glucose considered (10 µM in this case), where σ is the standard deviation for the same concentration (as shown in Figure 7b). The theoretical limit of quantification for our biosensor was estimated to be three times the detection limit.

## 4. Conclusions

A paradigm shift in the design and development of novel working electrodes for carrying out electrochemical studies has been demonstrated in this work, wherein ZnO nanostructures were grown on micropipette tips using a hydrothermal method. The developed working electrode using micropipette tips was characterized for its electrochemical properties and was subsequently used for glucose detection, to demonstrate a proof-of-concept for the use of such an electrode in electrochemical biosensing. The benefits of the developed micropipette tip-based biosensor are promising and include a linear range of 10–200 µM, sensitivity of 69.02 µA µM^−1^ cm^−2^, and a low detection limit of 67.5 µM. Furthermore, such modified micropipette tips could potentially act as cheaper versions of the conventional disk-based electrodes currently used in electrochemical studies. The current work is thus a significant step towards the integration of a three-electrode electrochemical system onto a single micropipette tip, as part of the miniaturization of electrochemical components, to achieve detection of various bio-analytes directly from the sample solution.

## Figures and Tables

**Table 1:** Electrochemical parameters calculated for the modified micropipette tips that were used as working electrodes.

Electrode Type	Anodic Potential (E_a_)	Cathodic Potential (E_c_)	Anodic Overpotential (η_a_)	Cathodic Overpotential (η_c_)	Gibbs Free Energy (ΔG)	Equilibrium Rate Constant (K_s_)
Ag-sputtered working electrode	0.799 V	−0.820 V	−0.192 V	0.213 V	~312 kJ/mol	3.880
ZnO-modified working electrode	0.660 V	−0.680 V	0.259 V	−0.279 V	~517 kJ/mol	3.811

**Table 2:** Comparison of the present work with the available literature.

Base Electrode + Biosensor Components	Electrochemical Method Adopted	Linear Range	Detection Limit	Sensitivity	Ref.
Gold electrode + ZnO nanowires/Nafion + Glucose oxidase	Amperometry (Enzymatic)	1–760 µM	700 µM	26.3 µA mM^−1^ cm^−2^	[19]
Gold electrode + electrodeposited ZnO nanotubes/Nafion + Glucose oxidase	Amperometry (Enzymatic)	50 µM–12 mM	1 µM	21.7 µA mM^−1^ cm^−2^	[43]
Indium Tin oxide electrode + ZnO nanorods/Nafion + Glucose oxidase	Amperometry (Enzymatic)	Not reported	0.22 µM	10.9 mA mM^−1^ cm^−2^	[16]
Glassy carbon electrode + ionic liquid/ZnO nanoparticles/egg-shell membrane + Glucose oxidase	Cyclic Voltammetry (Enzymatic)	1 pM–0.5 mM	0.1 pM	Not reported	[17]
Gold electrode + electrospun ZnO nanofibers/poly(vinyl alcohol) + L-cysteine + Glucose oxidase	Amperometry (Enzymatic)	0.25–19 mM	1 µM	70.2 µA mM^−1^ cm^−2^	[18]
Platinum electrode + ZnO nanorods/chemically reduced Graphene film/Nafion + Glucose oxidase	Amperometry (Enzymatic)	0.2–1.6 mM	Not reported	17.6 µA mM^−1^ cm^−2^	[24]
Platinum electrode + NiO doped ZnO nanorods + Glucose oxidase	Amperometry (Enzymatic)	0.5–8.0 mM	2.5 µM	61.8 µA mM^−1^ cm^−2^	[25]
**Micropipette tip + Ag-sputtered + hydrothermally grown ZnO nanostructures**	**Amperometry** **(Non-enzymatic)**	**10–200 µM**	**67.5 µM**	**69.02 µA µM^−1^ cm^−2^**	**This work**

## Data Availability

Not applicable.
